# Development and expert appraisal of a lifestyle questionnaire for microbiome host metadata

**DOI:** 10.3389/fcimb.2026.1932122

**Published:** 2026-10-21

**Authors:** Andrea Rocchetti, Franca Gotta, Paolo Bottino, Daria Vay, Valentina Pizzo, Valerio Gugliotta, Alessandra Agnese Grossi, Valeria Besutti, Mario Bianchetti, Elisa Bona, Ignazio Castagliuolo, Marta Corbella, Antonio Gasbarrini, Antonella Marangoni, Luca Masucci, Maria Vittoria Mauro, Lorenza Putignani, Gianluca Quaranta, Maurizio Sanguinetti, Lorenzo Drago

**Affiliations:** 1 Clinical Microbiology and Virology Unit, IRCCS AOU SS Antonio e Biagio e Cesare Arrigo, Alessandria, Italy; 2 Department of Science and Technological Innovation, University of Piemonte Orientale, Novara, Italy; 3 Department of Human Sciences and Innovation for the Territory (DISUIT), University of Insubria, Varese, Italy; 4 Research Center for Clinical Ethics (CREC), Department of Biotechnologies and Life Sciences, University of Insubria, Varese, Italy; 5 Microbiology Unit, University Hospital Padova, Padova, Italy; 6 Division of Gastroenterology, San Giuseppe Hospital, Gruppo Multimedica, Milan, Italy; 7 Department of Sustainable Development and Ecological Transition, University of Piemonte Orientale, Vercelli, Italy; 8 Center for Translational Research on Allergic and Autoimmune Diseases (CAAD), University of Piemonte Orientale, Novara, Italy; 9 Department of Molecular Medicine, University of Padova, Padova, Italy; 10 Microbiology and Virology Department, Fondazione IRCCS Policlinico San Matteo, Pavia, Italy; 11 CEMAD Digestive Disease Center, Fondazione Policlinico Universitario Agostino Gemelli IRCCS, Università Cattolica del Sacro Cuore, Roma, Italy; 12 Microbiology, Department of Medical and Surgical Sciences, University of Bologna, Bologna, Italy; 13 Department of Laboratory and Hematological Sciences, Fondazione Policlinico Universitario A. Gemelli Istituto di Ricovero e Cura a Carattere Scientifico (IRCCS), Rome, Italy; 14 Department of Basic Biotechnological Sciences, Intensive and Perioperative Clinics, Università Cattolica del Sacro Cuore, Rome, Italy; 15 Microbiology and Virology Department, AO Cosenza, Cosenza, Italy; 16 Unit of Microbiomics and Unit of Research of Microbiome, Bambino Gesù Children’s Hospital, IRCCS, Rome, Italy; 17 Department of Life Science, Health, and Health Professions, Link Campus University, Rome, Italy; 18 UOC Laboratory of Clinical Medicine with Specialized Areas, IRCCS MultiMedica, Milan, Italy; 19 Clinical Microbiology and Microbiome Laboratory, Department of Biomedical Sciences for Health, University of Milan, Milan, Italy

**Keywords:** FMT, lifestyle, microbiota, proof of concept, questionnaire development

## Abstract

**Introduction:**

Lifestyle information can help contextualize variation in the human gut microbiome, but its collection and operational definitions differ across studies. We developed a candidate questionnaire combining diet, physical activity and sleep for microbiome host metadata and undertook an initial appraisal by Italian experts. Candidate content was informed by a focused narrative literature appraisal and established lifestyle-assessment frameworks.

**Methods:**

Seventeen experts were invited and nine responded (53%). In a single anonymous rating round, experts assessed the relevance of 37 candidate items on a five-point scale. The prespecified agreement criterion was at least 70% of respondents assigning a rating of 4 or 5, corresponding to at least seven of nine experts.

**Results:**

Twenty-five items (67.6%) met this criterion: 18 of 23 dietary items (78.3%), six of eight physicalactivity items (75.0%), and one of six sleep items (16.7%). Twelve items remained exploratory.

**Discussion:**

These findings provide an initial prioritization of candidate lifestyle metadata within an Italian specialist network. They do not establish a stable iterative Delphi consensus, instrument reliability, construct validity, or associations between questionnaire responses and microbiome outcomes. Participant testing, operational refinement and empirical evaluation are required before research deployment or potential use in fecal microbiota transplantation donor characterization.

## Introduction

The human gut microbiota is a complex microbial ecosystem that plays a fundamental role in host physiology, including immune regulation, metabolic homeostasis and resistance to pathogens (Qin et al., 2010; Ursell et al., 2012; Sender et al., 2016). Advances in next-generation sequencing technologies have substantially expanded our understanding of microbiota diversity and metabolic potential, revealing substantial interindividual variability in microbiota composition (Turnbaugh et al., 2007; Fan and Pedersen, 2021).

Diet, physical activity and sleep are candidate exposures of interest in microbiome research, but the strength and specificity of the evidence differ across domains. Dietary patterns and fermentable substrates have been associated with microbial composition and metabolism; an increase in overall diversity is not a universal response to dietary fiber (Singh et al., 2017; So et al., 2018; Valdes et al., 2018; Baxter et al., 2019; Zmora et al., 2019). Studies of exercise report associations and intervention responses that vary with participant characteristics, diet and study design (Allen et al., 2018; Mailing et al., 2019; Clauss et al., 2021; Houttu et al., 2021; Moitinho-Silva et al., 2021). Circadian rhythms and sleep may also relate to microbial features, although human evidence remains limited and heterogeneous (Thaiss et al., 2014; Smith et al., 2019; Grosicki et al., 2020; Liu et al., 2020; Matenchuk et al., 2020). These findings justify recording relevant exposures without implying that every individual questionnaire item is an established causal determinant.

The collection and definition of lifestyle metadata vary across microbiome studies, which can complicate comparisons and interpretation of confounding (Falony et al., 2016; Zhernakova et al., 2016; Johnson et al., 2019; Vujkovic-Cvijin et al., 2020; Asnicar et al., 2021). In fecal microbiota transplantation (FMT), donor assessment primarily addresses safety, clinical eligibility and infectious risk; a survey of clinical and research practice has also documented variation in dietary assessment (Cammarota et al., 2019; Clancy et al., 2021; He et al., 2021). A lifestyle module could complement research characterization of donors, but its contribution to donor selection or treatment outcomes has not been established.

A growing body of microbiome epidemiology literature has identified host-related variables such as diet, medication use, physical activity, and environmental exposures as important sources of variation and potential confounding in human microbiome research. Large-scale microbiome studies have shown that these factors are associated with microbiota composition and metabolic function (Falony et al., 2016; Zhernakova et al., 2016; Johnson et al., 2019; Vujkovic-Cvijin et al., 2020; Asnicar et al., 2021). Variability in these host determinants can substantially influence microbiota profiles, thereby complicating the interpretation of microbiome datasets and limiting reproducibility across studies.

Existing instruments generally assess individual lifestyle domains, particularly diet, physical activity, or sleep, or are designed for specific clinical purposes. The GLAM Lifestyle Questionnaire was developed as an Italian proof-of-concept to integrate multiple lifestyle-related dimensions within a single structured host-metadata framework for microbiome research. At this stage, it should not be interpreted as a universally validated instrument; application outside the Italian context will require formal translation, cultural adaptation, cognitive testing, and population-specific validation.

Although developed and intended for initial validation within the Italian context, the questionnaire content was informed by international microbiome literature and by established epidemiological lifestyle-assessment tools, including the Food Frequency Questionnaire (FFQ). Cultural context should therefore not be regarded as a neutral background variable in lifestyle assessment, but as an intrinsic component of how lifestyle exposures are expressed, reported, and ultimately related to the microbiome; it must consequently be made explicit when interpreting the instrument and when adapting it to different populations.

The methodological gap addressed here is the incomplete and inconsistent collection of standardized lifestyle-related host metadata alongside microbiome measurements. This gap makes it harder to interpret differences between individuals and to compare findings across cohorts. Cultural context affects how lifestyle exposures are expressed and reported, and therefore needs to be documented when an instrument is applied or adapted. A structured survey covering diet, physical activity, and sleep may help collect such metadata consistently. Accordingly, this study aimed to develop and undertake preliminary expert content evaluation of a lifestyle questionnaire for microbiome epidemiology, with potential future application to FMT donor characterization. Its performance in participants and its transferability across cultural settings remain to be tested.

## Methods

### Study design

This methodological proof-of-concept study developed a candidate lifestyle questionnaire and assessed the relevance of its content in a single anonymous expert-rating round. The exercise was initially described as a modified Delphi process, but it did not include iterative voting or controlled feedback and is therefore reported here as an initial expert appraisal. Development comprised a focused narrative literature appraisal, identification of candidate lifestyle variables, questionnaire preparation, and expert relevance ratings. No participant-level lifestyle or microbiome outcomes were collected.

Development and expert appraisal were conducted in an Italian scientific context. Subsequent participant testing is planned initially in Italy before any wider application.

### Literature review and identification of lifestyle variables

A focused narrative literature appraisal informed candidate-variable development across diet, physical activity and sleep. Searches in PubMed (MEDLINE) and Scopus were reported during development; the available notes document PubMed searches, including searches for existing domain-specific questionnaires. Complete dated exports, the exact Scopus search history and record-level selection decisions were not retained in the available materials. Thematic concepts combined gut microbiota or microbiome with diet or nutrition, physical activity or exercise, and sleep or circadian rhythm. **Box 1** summarizes the scope of the evidence considered, not a reproducible historical screening protocol. Human observational and intervention studies and reviews provided contextual rationale, not a pooled estimate or evidence of a causal effect for every variable.

Box 1 **Scope of the literature appraisal for item generation.**

**Table:** 

Domain	Sources considered for domain rationale	Outside the exposure-evidence scope
*Population/model*	Studies conducted on human subjects	Studies based on animal or *in vitro* models
*Exposure of interest*	Studies investigating lifestyle factors, specifically diet, physical activity, or sleep	Studies not addressing lifestyle determinants of gut microbiota
*Outcome of interest*	Studies examining gut microbiota composition or metabolic function, with sufficient data on microbiota–lifestyle interactions	Studies focusing on microbiota unrelated to the gastrointestinal tract, such as oral or vaginal microbiota
*Publication type*	Original research articles and review papers	Editorials, commentaries, and case reports without systematic data
*Language*	Articles published in English	Articles not available in English

This was a focused narrative appraisal rather than an exhaustive systematic review. A complete, deduplicated record set and decisions for individual full texts were not available for reconstruction of a study-selection flow. The publications that could be identified were mapped to candidate items to distinguish exposure-related evidence, broader context, measurement frameworks and gaps in direct evidence. The resulting map describes the rationale for item development; it does not establish the membership of a historical screened set.

The initial literature appraisal and candidate-variable selection were performed manually. The original development protocol did not include AI-, NLP-, or machine-learning-assisted screening. This narrative appraisal was intended to identify representative evidence for questionnaire development rather than to constitute a systematic review or quantitative synthesis.

### Questionnaire design

The questionnaire was developed within the GLAM Working Group (Gut Microbiota Study Group) of the Italian Association of Clinical Microbiologists (AMCLI), a national multidisciplinary network dedicated to microbiota research and microbiome-based clinical applications. The project was initiated by the Clinical Microbiology and Virology Unit of the Azienda Ospedaliero-Universitaria SS. Antonio e Biagio e Cesare Arrigo in Alessandria, Italy, a center hosting a microbiome sequencing facility and authorized by the Italian National Transplant Center for FMT.

Questionnaire content was informed by the narrative literature appraisal and established lifestyle-assessment frameworks, including dietary assessment, the International Physical Activity Questionnaire (IPAQ) and the Pittsburgh Sleep Quality Index (PSQI) (Buysse et al., 1989; Craig et al., 2003; Willett, 2013). The resulting 37 candidate items covered diet (Hsu and Sandford, 2007), physical activity (Singh et al., 2017) and sleep (Valdes et al., 2018), as detailed in **Tables 1**–**3**. Established measurement properties of the source frameworks cannot be transferred to the GLAM items or an overall GLAM score.

**Table 1:** Dietary lifestyle variables included in the questionnaire.

Item	Variable	Experts rating 4–5 n (%)	Criterion met	Candidate relevance and evidence limit
1	Fruit consumption frequency	8/9 (88.9)	Yes	Fruit intake provides dietary context; human associations do not establish a causal item effect (Partula et al., 2019; Yu et al., 2021; Maukonen et al., 2024).
2	Vegetable consumption frequency	8/9 (88.9)	Yes	Plant-food associations differ by food group and population (Yu et al., 2021; Maukonen et al., 2024).
3	Whole grain intake	8/9 (88.9)	Yes	Grain and fiber context; whole-grain effects are not isolated in every source (So et al., 2018; Houttu et al., 2021; Maukonen et al., 2024).
4	Dietary fiber intake	9/9 (100)	Yes	Fiber interventions affect some taxa or metabolites; diversity does not invariably increase (So et al., 2018; Baxter et al., 2019).
5	Legume consumption	7/9 (77.8)	Yes	Legumes are assessed with other plant foods; legume-specific effects are not isolated (So et al., 2018; Yu et al., 2021).
6	Fermented food consumption	7/9 (77.8)	Yes	Fermented dairy and food-associated microbes provide context; findings cannot be generalized to all fermented foods (Maukonen et al., 2024; Fackelmann et al., 2025).
7	Dairy product consumption	7/9 (77.8)	Yes	Dairy associations vary by product and microbial outcome (Partula et al., 2019; Yu et al., 2021; Maukonen et al., 2024).
8	Red meat consumption	7/9 (77.8)	Yes	Red-meat and animal-food associations provide context; a universal TMAO mechanism is not established (Maukonen et al., 2024; Fackelmann et al., 2025).
9	Processed meat consumption	7/9 (77.8)	Yes	Processed-meat associations remain observational and potentially confounded (Partula et al., 2019; Yu et al., 2021; Maukonen et al., 2024).
10	Fish consumption	7/9 (77.8)	Yes	Fish is assessed in dietary cohorts; an omega-3 mechanism is not established for this item (Partula et al., 2019; Yu et al., 2021).
11	Sugary beverage intake	7/9 (77.8)	Yes	Sugary-drink associations are observational and do not establish causal dysbiosis (Partula et al., 2019).
12	Ultra-processed food consumption	8/9 (88.9)	Yes	Prepared-food and diet-quality findings provide indirect context; a NOVA-defined effect is not established here (Partula et al., 2019; Yu et al., 2021).
13	Alcohol consumption	7/9 (77.8)	Yes	Proposed alcohol-exposure metadata; an independent microbiota effect is not established by this appraisal.
14	Fast-food consumption frequency	7/9 (77.8)	Yes	Food-group and dietary-pattern findings provide indirect context; fast-food frequency is not independently validated (Partula et al., 2019).
15	Daily water intake	7/9 (77.8)	Yes	Human water-intake associations support recording exposure; observational categories do not validate a GLAM volume threshold (Vanhaecke et al., 2022).
16	Probiotic food consumption	7/9 (77.8)	Yes	Food-associated microbes provide context; probiotic efficacy of this item is not established (Maukonen et al., 2024; Fackelmann et al., 2025).
17	Prebiotic-rich food consumption	8/9 (88.9)	Yes	Fermentable-substrate evidence supports recording exposure; foods, dose and outcomes need definition (So et al., 2018; Baxter et al., 2019).
18	Plant-based food consumption	8/9 (88.9)	Yes	Diet-pattern evidence is heterogeneous; no universal increase in diversity is established (Kohnert et al., 2021; Yu et al., 2021; Fackelmann et al., 2025).
19	Type of drinking water	6/9 (66.7)	No	Human water-source associations support recording exposure; they do not establish causation or item validity (Vanhaecke et al., 2022).
20	Organic food consumption	6/9 (66.7)	No	A small organic-versus-conventional dietary study provides preliminary context; findings do not establish general superiority of organic foods (Di Renzo et al., 2025).
21	Cooking methods	6/9 (66.7)	No	Food categories provide indirect context; cooking method is not isolated from food composition (Partula et al., 2019).
22	Type of sweeteners used	6/9 (66.7)	No	A human trial supports recording specific non-nutritive sweeteners; effects vary by sweetener and person, and do not validate GLAM wording (Suez et al., 2022).
23	Food purchasing location	5/9 (55.6)	No	Proposed purchasing-context metadata; a location-specific microbiota association was not identified in this focused appraisal.

**Table 2:** Physical activity variables included in the questionnaire.

Item	Variable	Experts rating 4–5 n (%)	Criterion met	Candidate relevance and evidence limit
24	Frequency of vigorous physical activity	8/9 (88.9)	Yes	Exercise context and activity-assessment framework; diet and participant differences require consideration (Craig et al., 2003; Allen et al., 2018).
25	Duration of vigorous activity	7/9 (77.8)	Yes	Training-duration context supports recording exposure, without establishing a dose-response threshold (Craig et al., 2003; Allen et al., 2018).
26	Frequency of moderate physical activity	6/9 (66.7)	No	Moderate-activity context and measurement framework; microbial responses are heterogeneous (Craig et al., 2003; Houttu et al., 2021; Moitinho-Silva et al., 2021).
27	Walking frequency	6/9 (66.7)	No	Activity-assessment component; a walking-specific microbiota association is not established in the mapped sources (Craig et al., 2003).
28	Total weekly physical activity time	8/9 (88.9)	Yes	Habitual activity provides exposure context; total weekly time is not a validated independent microbiota predictor (Craig et al., 2003; Houttu et al., 2021).
29	Sedentary behavior (daily sitting time)	7/9 (77.8)	Yes	Active–sedentary context; daily sitting time is not an established independent microbiota predictor (Craig et al., 2003; Houttu et al., 2021).
30	Participation in structured exercise	7/9 (77.8)	Yes	Exercise interventions report variable responses; the item does not imply a uniform microbial effect (Allen et al., 2018; Moitinho-Silva et al., 2021).
31	Occupational physical activity	7/9 (77.8)	Yes	Activity-assessment context; occupation-specific microbiota associations are not established here (Craig et al., 2003).

**Table 3:** Sleep-related variables included in the questionnaire.

Item	Variable	Experts rating 4–5 n (%)	Criterion met	Candidate relevance and evidence limit
32	Total hours of sleep per night	7/9 (77.8)	Yes	Sleep–microbiota associations provide context; small or observational studies do not validate this item (Smith et al., 2019; Matenchuk et al., 2020).
33	Sleep onset latency	5/9 (55.6)	No	Sleep-assessment component; a latency-specific microbiota association is not established (Buysse et al., 1989; Grosicki et al., 2020).
34	Wake-up time	5/9 (55.6)	No	Sleep–wake timing context; wake-up time alone is not isolated (Buysse et al., 1989; Liu et al., 2020).
35	Subjective sleep quality	5/9 (55.6)	No	Pilot global sleep-quality associations provide context; GLAM wording is not independently validated (Buysse et al., 1989; Grosicki et al., 2020).
36	Night awakenings	5/9 (55.6)	No	Sleep-fragmentation associations provide context, without causal item-level validation (Buysse et al., 1989; Smith et al., 2019).
37	Use of sleep medication	4/9 (44.4)	No	Sleep-assessment component and potential medication covariate; adjustment for medication use is not evidence of a drug-specific microbiota effect (Buysse et al., 1989; Estaki et al., 2023).

The 37 items represent candidate content for lifestyle metadata rather than a finalized measurement scale. Their relevance to research was rated by experts; comprehensibility for respondents, completeness of coverage, response categories, recall periods and measurement performance were not evaluated in this phase. No composite lifestyle score was developed or validated.

### Expert relevance appraisal

The GLAM Working Group circulated the candidate questionnaire within its national specialist network for an anonymous expert appraisal. The objective was to prioritize content potentially relevant to microbiome research. The exercise used independent ratings and a prespecified agreement criterion but did not include feedback between rounds.

The questionnaire was circulated to 17 experts within the GLAM network, selected on the basis of documented experience in microbiota research, microbiome diagnostics, FMT, or related clinical and translational fields. The survey was administered online by the study coordinator between September and October 2025 via Google Forms. Of the 17 invited experts, 9 completed the consensus assessment (participation rate, 53%) and constituted the expert panel used for the quantitative consensus analysis.

The panel included expertise in clinical microbiology, microbiota research, infectious diseases, gastroenterology and microbial genomics. The participating experts are described in **Table 4**; authorship and membership of the wider GLAM network are not treated as evidence of participation in the formal ratings.

**Table 4:** Professional background and specific expertise of the nine experts participating in the expert relevance appraisal.

Expert	Professional background/institutional setting	Main expertise relevant to the expert appraisal
E1	Clinical microbiologist, university hospital	Clinical microbiology; FMT; microbiota banking and quality/stability of FMT products
E2	Associate Professor, university	Human microbial ecology; gut microbiota; metagenomics/metabarcoding; diet–microbiota interactions
E3	Associate Professor of Microbiology and Clinical Microbiology, university	Clinical microbiology; human microbiome; gut dysbiosis; probiotics and microbiota-related research
E4	Full Professor of Infectious Diseases, university	Infectious diseases; gut microbiome; host–microbiota interactions; intestinal dysbiosis and clinical translation
E5	Associate Professor of Microbiology and Clinical Microbiology, university	Human microbiota; vaginal microbiota; clinical microbiology; FMT and stool banking
E6	Clinical microbiologist and researcher, university hospital/academic center	Clinical microbiology; gut microbiota; FMT; stool banking and donor screening
E7	Clinical microbiologist, university hospital	Gut microbiota; FMT; donor screening; microbiota manipulation and laboratory procedures
E8	Director of Clinical Microbiology and Virology, university hospital	Clinical microbiology; gut microbiota; FMT; metagenomics/NGS and clinical implementation
E9	Clinical microbiologist, university hospital	Clinical microbiology; FMT laboratory procedures and stool banking; gut microbiota research

**Table 4** summarizes the professional setting and expertise of the nine respondents, identified as E1–E9.

The participation rate was 9/17 (53%); all item-level agreement percentages refer to the nine respondents, not to the full invited group.

All experts completed the survey independently and responses were collected anonymously to minimize the risk of mutual influence among panel members. Each questionnaire item was independently evaluated on a 5-point Likert scale assessing its scientific relevance to microbiota research, where 1 indicated “not relevant”, 2 “slightly relevant”, 3 “moderately relevant”, 4 “relevant”, and 5 “highly relevant”.

The agreement criterion was prespecified as at least 70% of respondents assigning an item a rating of 4 or 5, consistent with the range of thresholds used in healthcare consensus studies (Hsu and Sandford, 2007; Keeney et al., 2011; Diamond et al., 2014). With nine respondents, this required at least seven positive ratings (7/9, 77.8%); six positive ratings corresponded to 66.7% and did not meet the criterion. Agreement describes the panel’s relevance judgment and is not a grade of evidence certainty.

Items meeting the criterion were designated consensus-supported candidate items; those below it were retained as exploratory. This designation describes perceived research relevance and does not imply measurement validity or a demonstrated association with microbiome outcomes. No items or ratings were retrospectively changed.

The single rating round establishes initial agreement within this panel. Without iterative feedback or repeat voting, the stability of that agreement cannot be assessed, and the study should not be interpreted as a complete conventional Delphi exercise (Gattrell et al., 2024).

The prespecified criterion was retained without *post hoc* adjustment. Further expert rounds with controlled feedback, including reassessment of borderline items, would be needed to evaluate agreement stability.

### Study outcomes

The primary outcome was the proportion of candidate items meeting the prespecified expert agreement criterion for relevance to microbiome research.

Secondary outcomes were the distribution of agreement across diet, physical activity and sleep, and the identification of below-threshold items for further development.

Item-level results are presented as aggregate counts and percentages of experts assigning ratings of 4 or 5. The counts in **Tables 1**–**3**, **5** are arithmetically consistent with the 7/9 threshold and domain totals. Individual responses were unavailable for independent reanalysis, so secondary medians and quartiles are not presented; the limitation of the available numerical record is detailed below.

**Table 5:** Status of questionnaire items after expert appraisal.

Domain	Total items	Items meeting criterion	Items below criterion	Criterion met (%)	Interpretation
*Diet*	23	18	5	78.3%	Largest proportion meeting the criterion in this panel
*Physical activity*	8	6	2	75.0%	Six of eight candidate items met the criterion
*Sleep*	6	1	5	16.7%	One of six candidate items met the criterion
*Total*	37	25	12	67.6%	Initial relevance prioritization within this expert panel

### Ethical considerations

The study involved voluntary expert consultation about the relevance of candidate questionnaire content. It did not recruit patients or collect biological samples or personal health information. Experts were informed of the survey purpose; completion was treated as agreement to participate. Formal ethics review was not sought because the exercise was considered an anonymous, non-clinical professional consultation.

## Results

Of the 37 candidate items, 25 (67.6%) met the prespecified agreement criterion and 12 (32.4%) remained exploratory. These are relevance ratings by the responding experts and do not constitute empirical microbiome findings.

Dietary items showed the highest proportion meeting the criterion: 18/23 (78.3%). These included fruit, vegetables, grains, fiber, fermented foods and other dietary descriptors. Five dietary items remained exploratory: type of drinking water, organic food consumption, cooking methods, type of sweeteners and food purchasing location.

Six of eight physical-activity items (75.0%) met the criterion. The two below-threshold items, moderate-activity frequency and walking frequency, each received six positive ratings (66.7%).

One of six sleep items (16.7%), total sleep duration, met the criterion. Sleep onset latency, wake-up time, subjective sleep quality, night awakenings and use of sleep medication remained exploratory.

Among the 25 items meeting the criterion, 16 received seven positive ratings, eight received eight, and one received nine. Across all three domains, six of the 12 below-threshold items received six positive ratings. Each expert represents 11.1 percentage points with this panel size; the classification of items near the threshold should therefore be interpreted cautiously.

**Table 5** summarizes domain-level results; **Tables 1**–**3** present all 37 candidate variable names, aggregate positive-rating counts and agreement classifications. The candidate questionnaire should be read as content undergoing development rather than a research instrument ready for deployment.

The explanatory column in **Tables 1**–**3** summarizes candidate relevance and evidence limits using a narrative appraisal, separate from the expert ratings. No formal grades of evidence certainty are assigned because the literature was not assessed with a prespecified grading procedure or systematic risk-of-bias assessment. Some items were proposed as contextual metadata despite limited direct human microbiota evidence. References to established assessment instruments indicate measurement context, not validation of GLAM wording.

### Interpretation of expert agreement

The expert appraisal prioritized candidate metadata across three lifestyle domains. Higher agreement on diet indicates the preferences of this panel; it does not establish stronger item-level causal evidence or justify excluding sleep and physical-activity exposures. The preliminary module is intended to support future harmonized data collection, but its contribution to microbiome interpretation requires empirical evaluation.

## Discussion

This study addresses an important methodological challenge in microbiome research, namely the interpretation of microbial sequencing data in the absence of standardized contextual information on host lifestyle and environmental exposures. Although prior studies have described donor-screening questionnaires used in FMT, lifestyle surveys embedded in donor assessment, and domain-specific instruments relevant to microbiota research, such as microbiota-focused dietary questionnaires, the novelty of the present work lies primarily in integrating multiple lifestyle domains into a single structured framework specifically developed for microbiome research and subjected to initial expert relevance appraisal, rather than in the individual questionnaire domains themselves (van Nood et al., 2013; Allegretti et al., 2019; Formisano et al., 2024). This need is further underscored by evidence that donor dietary patterns are not routinely incorporated into FMT screening practice (Clancy et al., 2021). Importantly, real-world donor-screening programs have demonstrated low eligibility rates and require complex, multistep evaluation processes integrating clinical, behavioral, and microbiological criteria, further supporting the potential value of standardized tools for donor characterization (Bénard et al., 2022).

The geographical and cultural scope of the present work also requires clarification. Both questionnaire development and the formal expert assessment were conducted in Italy, and the current instrument therefore reflects, at least in part, an Italian/Western European dietary and lifestyle context. Examples such as fermented foods, polyphenol-rich foods, and food-purchasing patterns may differ in prevalence, meaning, and microbiome relevance across cultures. The next validation phase is planned in Italy, where the population is increasingly multicultural and characterized by heterogeneous dietary and lifestyle patterns; this will provide an initial opportunity to assess comprehensibility and applicability across a diverse national population. Nevertheless, Italian validation cannot establish universal applicability, and international use will require formal translation, cultural adaptation, cognitive testing, and validation in the relevant target populations.

The panel prioritized a larger proportion of dietary than sleep-related items. Diet–microbiota research provides a rationale for this domain, but panel agreement cannot confirm biological effects or establish that every selected food descriptor has an independent microbiome association (Singh et al., 2017; So et al., 2018; Partula et al., 2019; Zmora et al., 2019; Kohnert et al., 2021; Yu et al., 2021; Maukonen et al., 2024; Fackelmann et al., 2025).

Physical-activity items were also prioritized, consistent with the relevance of recording exercise exposure. Published associations and intervention responses are heterogeneous, and participant characteristics, dietary differences and baseline microbial features can affect interpretation (Allen et al., 2018; Mailing et al., 2019; Clauss et al., 2021; Houttu et al., 2021; Moitinho-Silva et al., 2021).

The lower agreement on sleep-related items may reflect the panel’s knowledge, item formulation or uncertainty about research relevance; this study cannot distinguish these explanations. Human sleep–microbiota findings provide a rationale for further investigation, including exposures below the present agreement threshold (Thaiss et al., 2014; Smith et al., 2019; Grosicki et al., 2020; Liu et al., 2020; Matenchuk et al., 2020).

From an ecological perspective, the human microbiota can be understood as a dynamic ecosystem shaped by multiple environmental and behavioral influences. Lifestyle-related determinants captured through structured surveys may be viewed as measurable components of the human exposome that contribute to gut microbiota composition and interindividual variability (Johnson et al., 2019; Vujkovic-Cvijin et al., 2020). Rather than being interpreted as direct causal determinants of specific microbial configurations, these variables should be intended as environmental and behavioral factors that help explain microbiota variability across individuals. In this sense, the proposed questionnaire can therefore be interpreted as a candidate core lifestyle module within broader host metadata collection strategies.

The framework underlying the proposed questionnaire links lifestyle exposures, microbial diversity and metabolism, and host physiological processes such as immune regulation and metabolic homeostasis (Vujkovic-Cvijin et al., 2020; Asnicar et al., 2021; Fan and Pedersen, 2021). These relationships provide a rationale for collecting host metadata, without demonstrating a causal pathway for individual items or effects on microbiota-based therapies.

Within this perspective, the development of a structured lifestyle survey represents an important preliminary methodological step for microbiome research. Standardized surveys are designed to facilitate the systematic collection of host metadata that are otherwise difficult to capture in sequencing-based studies. Rather than defining rigid diagnostic categories, their objective is to identify contextual patterns that may help interpret microbiota variability and facilitate comparisons across studies (Vujkovic-Cvijin et al., 2020). Empirical validation of the questionnaire will require its application in appropriately powered microbiome cohorts, enabling assessment of associations between lifestyle variables and microbial composition, diversity, and function (Falony et al., 2016; Zhernakova et al., 2016).

Expert relevance appraisal can help prioritize candidate content when evidence and measurement needs are heterogeneous (Hsu and Sandford, 2007; Keeney et al., 2011; Diamond et al., 2014). It does not resolve uncertainty in the underlying literature. Subsequent iterative consensus work should explicitly report panel selection, feedback, decisions and stopping rules, in line with consensus-reporting guidance (Gattrell et al., 2024).

Lifestyle assessment may also have implications for microbiota-based therapeutic approaches, particularly FMT. Current donor screening protocols primarily focus on safety considerations and infectious risk; however, donor microbiota characteristics may influence therapeutic outcomes, as suggested by the “super-donor” phenomenon (Wilson et al., 2019). The systematic collection of lifestyle information may therefore provide additional insights into donor microbiota composition and functional potential, contributing to improved donor characterization strategies (Clancy et al., 2021).

### Strengths and limitations

This study has several strengths. The questionnaire was developed within a clinical microbiology setting integrating microbiome sequencing activities and an FMT program, providing a clinically relevant context for its development across research and microbiota-oriented applications. As a candidate modular tool, it is intended to facilitate harmonized data collection in multicenter observational studies, longitudinal cohorts, and interventional studies targeting dietary or metabolic modulation. Candidate variables could potentially complement research characterization of FMT donors; however, administration time, burden, feasibility, and acceptability require empirical assessment in the target population.

More broadly, the questionnaire can be conceptualized as a lifestyle module within a wider host metadata framework, to be integrated with pharmacological exposures, environmental factors, clinical and metabolic parameters, and sociodemographic characteristics. Its modular design may support future scalability in multicenter settings and potential integration into digital data collection platforms and clinical information systems, subject to empirical evaluation of feasibility and acceptability. In this context, it may represent a proof-of-concept tool for future collaborative microbiome research. The integration of standardized lifestyle metadata with microbiome sequencing approaches, including 16S rRNA and metagenomics, may contribute to a more ecological interpretation of host–microbiota interactions and improve the understanding of microbiota variability across populations.

The scope of the proposed questionnaire is limited to lifestyle-related variables and does not provide a comprehensive assessment of the exposome. Other determinants of microbiome variability, including antibiotic and medication exposure, psychological and socioeconomic factors, geographic mobility and travel, household and pet exposures, and occupational or environmental factors, are more appropriately considered within a broader host-exposure framework. A subsequent phase of the GLAM research program is intended to explore these wider dimensions and their integration with microbiome data, without treating them as part of the 37 items appraised in this study.

Established health-behavior frameworks could inform later development of explanatory modules, but any added constructs would require separate item development and evaluation (Janz and Becker, 1984; Bosnjak et al., 2020; Rubinelli and Diviani, 2020).

The expert panel was relatively small and was drawn from a national specialist network, with 9 of the 17 invited experts completing the formal consensus assessment (53%). This participation rate introduces the possibility of non-response bias, as non-participating experts may have differed in their assessment of the proposed lifestyle variables. Consequently, the observed consensus may not fully represent the range of views within the broader microbiome community and should be interpreted as preliminary. Future validation should involve a larger and more geographically and professionally diverse expert panel.

The single rating round lacked iterative feedback and reassessment, so agreement stability was not evaluated. The study assessed expert relevance only, not respondent comprehensibility, completeness of lifestyle coverage or measurement validity. The candidate questionnaire has not been administered to a target population. Feasibility, acceptability, recall periods, response options and repeatability require testing. Associations with microbial alpha and beta diversity, taxa and functional or metabolic profiles were not assessed. Further expert rounds and appropriately designed participant studies are needed.

The proposed questionnaire would collect self-reported lifestyle information. In subsequent participant studies, recall and social-desirability biases, differences in item interpretation and inaccurate quantification of habitual behaviors may introduce measurement error. Such errors could obscure exposure–microbiome associations; these potential effects were not measured in the present expert appraisal.

An additional limitation concerns the narrative literature appraisal used for variable generation. Complete database exports and record-level screening decisions were unavailable, precluding a verified study-selection flow or list of individual full-text exclusions. Searches in PubMed and Scopus were reported during development, but the available notes detail only PubMed searches and do not include a dated Scopus export. The bibliographic map improves visibility of the rationale but cannot establish historical retrieval, completeness of coverage, or a direct microbiota association for every variable. No systematic assessment of risk of bias or certainty of evidence was undertaken. The Italian composition of the expert panel may also have favored exposures especially salient in this context.

The numerical appraisal is limited by the absence of the original item-level responses for reanalysis. In the initial summary, five medians were mathematically incompatible with their positive-rating counts (items 23 and 33–36). We therefore report neither those medians nor secondary quartile summaries. Although the retained aggregate counts and 7/9 classifications are internally consistent, they could not be checked against the survey export. Reconciliation with the source responses would strengthen independent replication of the expert appraisal before the instrument is finalized.

Operational definitions remain preliminary. Expressions such as “regularly,” “frequently,” “often” and “adequate” require explicit recall periods and response categories before participant use. Cognitive interviewing, feasibility testing, test–retest assessment and comparison with appropriate exposure measures should precede microbiome association studies. Because diet, activity and sleep are different exposure domains, an overall GLAM score or a single internal-consistency coefficient should not be assumed appropriate without a defined measurement model. Subsequent wording changes to expert-rated items would require renewed assessment.

## Conclusions

This Italian proof-of-concept study developed a 37-item candidate lifestyle questionnaire and found that 25 items met a prespecified relevance-agreement criterion among nine experts. The contribution is an initial prioritization of content for microbiome host metadata. It does not establish instrument validity, agreement stability, microbiome associations or utility for FMT donor selection.

Next steps are operational refinement, cognitive testing, further expert appraisal and evaluation of exposure measurement in an Italian population. Microbiome association studies should then assess whether the collected metadata improve interpretation beyond clinical, pharmacological and demographic covariates. Wider use would require translation and cultural adaptation.

The candidate lifestyle module may subsequently be integrated within a broader host-exposure framework, while keeping future modifications distinct from the 37 items appraised in this study.

## Data Availability

The original contributions presented in the study are included in the **Supplementary Material**, further inquiries can be directed to the corresponding author/s.
