# Supplementary material for: Development and expert appraisal of a lifestyle questionnaire for microbiome host metadata
Source: Front Cell Infect Microbiol. 2026 Oct 21;16:1932122. doi: 10.3389/fcimb.2026.1932122 (PMC13730856; doi:10.3389/fcimb.2026.1932122)
Supplement: Supplementary file 1 [file DataSheet1.pdf]

Supplementary Material

Development and expert validation of a standardized lifestyle questionnaire for microbiome host metadata: a GLAM Working Group proof-of-concept study

Andrea Rocchetti, Franca Gotta, Paolo Bottino, Daria Vay, Valentina Pizzo, Valerio Gugliotta, Alessandra Agnese Grossi, Valeria Besutti, Mario Bianchetti, Elisa Bona, Ignazio Castagliuolo, Marta Corbella, Antonio Gasbarrini, Antonella Marangoni, Luca Masucci, Maria Vittoria Mauro, Lorenza Putignani, Gianluca Quaranta, Maurizio Sanguinetti, Lorenzo Drago

Supplementary Figure S1. PRISMA 2020-informed flow diagram of study selection

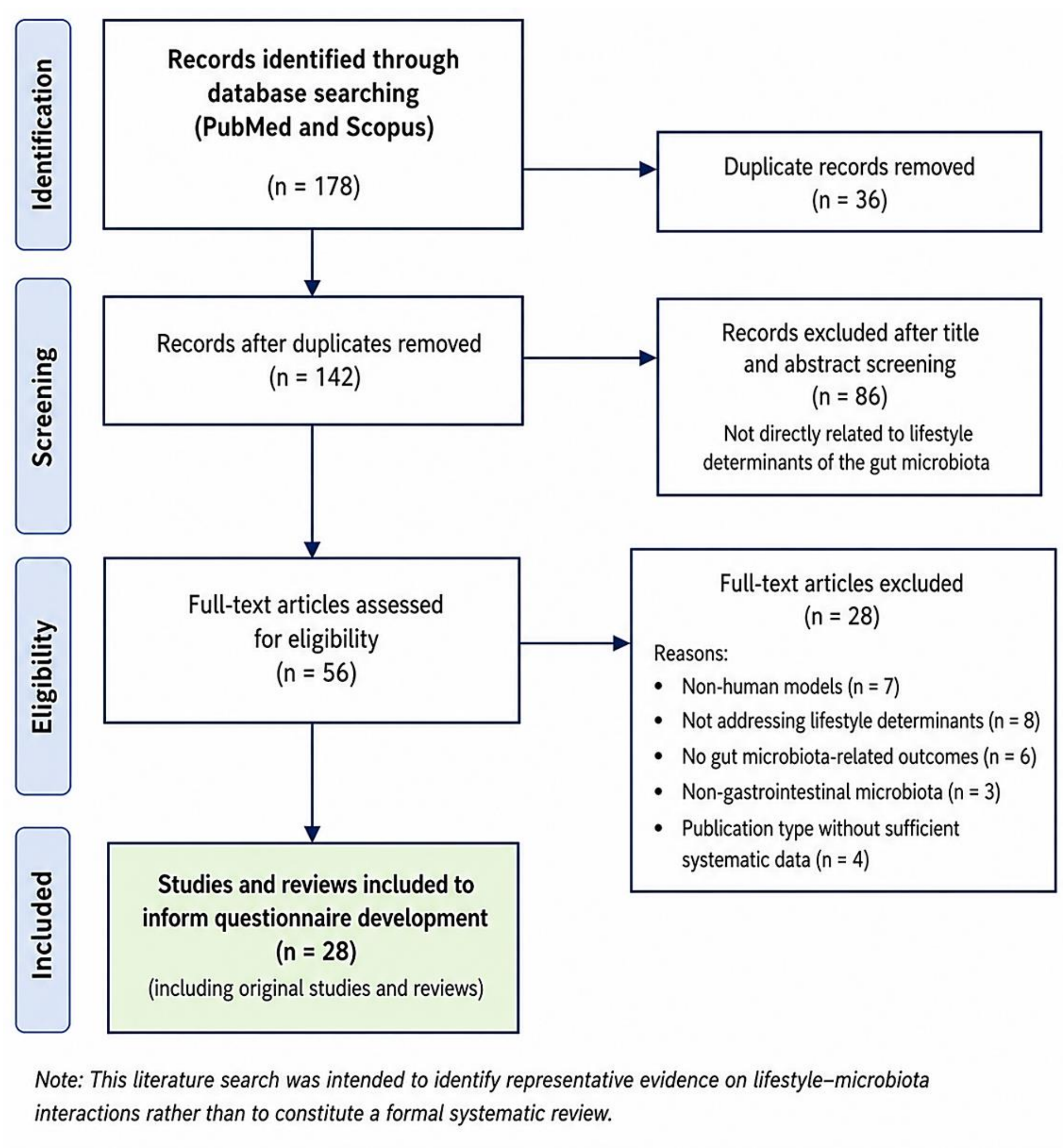

**Supplementary Table S1 – GLAM Lifestyle Questionnaire**	
Section 1 Dietary habits (Diet)	
1.	I consume fresh fruit regularly.
2.	I consume vegetables daily.
3.	Whole grains are a regular part of my diet.
4.	I frequently eat legumes (beans, lentils, chickpeas).
5.	I consume fermented foods (e.g., yogurt, kefir, kimchi).
6.	My diet includes fiber-rich foods.
7.	I frequently consume red meat.
8.	I consume processed meat products (e.g., sausages, salami).
9.	I regularly consume fish or seafood.
10.	Dairy products are part of my regular diet.
11.	I frequently eat ultra-processed foods.
12.	I drink sugary beverages regularly.
13.	I consume alcoholic beverages.
14.	I consume foods rich in polyphenols (e.g., berries, olive oil, dark chocolate).
15.	My diet is predominantly plant-based.
16.	I eat fast food.
17.	I consume snacks between meals.
18.	I consume foods with added sugars.
19.	I use artificial sweeteners.
20.	My primary drinking water source is bottled or filtered water.
21.	I preferentially consume organic foods.
22.	I frequently use cooking methods such as frying or grilling.
23.	I usually purchase food from supermarkets rather than local markets.
Section 2 — Physical activity	
24.	I perform physical activity regularly during the week.
25.	My exercise sessions are of moderate or high intensity.
26.	I practice aerobic activities (running, cycling, swimming).
27.	I perform strength or resistance training.
28.	I spend many hours sitting during the day.
29.	My work involves physical activity.
30.	I practice recreational physical activities.
31.	I walk regularly during the day.
Section 3 — Sleep habits	
32.	I usually sleep an adequate number of hours per night.
33.	I consider my sleep quality to be good.
34.	I fall asleep quickly.
35.	I wake up during the night.
36.	I maintain a regular sleep schedule.
37.	My sleep follows a regular circadian rhythm.
