# Supplementary material for: Development and expert appraisal of a lifestyle questionnaire for microbiome host metadata
Source: Front Cell Infect Microbiol. 2026 Oct 21;16:1932122. doi: 10.3389/fcimb.2026.1932122 (PMC13730856; doi:10.3389/fcimb.2026.1932122)
Supplement: Supplementary file 3 [file Table2.docx]

Appendix to the response to Reviewer 3

Retrospective bibliography and item development audit

This audit identifies sources in the available development bibliography (A01–A31) and additions made during revision (R01–R04). It is not a verified reconstruction of the former 28 included reports. Design and relevance were checked against the available bibliographic appraisal; study sample sizes and historical full-text eligibility were not systematically re-extracted. The table therefore does not invent sample-size or historical inclusion data.

# A Study characteristics and relevance

Item numbers identify retrospective relationships to the reported GLAM analytic inventory. Reviews are labelled and do not constitute separate participant samples. R sources were added during revision.

| **Source** | **Design or type** | **Contribution and limit** | **Item** |
| --- | --- | --- | --- |
| A01 Allen JM, Mailing LJ, Niemiro GM, Moore R, Cook MD, White BA, et al (2018) | Exercise intervention in humans | Exercise-associated composition and functional change; response may vary by baseline body composition. | 24, 25, 26, 30 |
| A02 Bressa C, Bailén-Andrino M, Pérez-Santiago J, González-Soltero R, Pérez M, Montalvo-Lominchar MG, et al (2017) | Active versus sedentary human comparison | Active/sedentary differences; observational comparison does not isolate exercise from other factors. | 29 |
| A03 Cerdá B, Pérez M, Pérez-Santiago JD, Tornero-Aguilera JF, González-Soltero R, Larrosa M (2016) | Narrative review | Exercise–microbiota background; review does not provide an independent participant sample. | Domain context |
| A04 Clarke SF, Murphy EF, O'Sullivan O, Lucey AJ, Humphreys M, Hogan A, et al (2014) | Athlete versus control comparison | Athlete-associated diversity; extreme diet and training are intertwined. | 24 |
| A05 Donati Zeppa S, Amatori S, Sisti D, Gervasi M, Agostini D, Piccoli G, et al (2021) | Before and after exercise intervention | Composition changed after training, while diversity did not clearly change. | 24, 25, 30 |
| A06 Dziewiecka H, Buttar HS, Kasperska A, Ostapiuk-Karolczuk J, Domagalska M, Cichoń J, et al (2022) | Systematic review | Summarizes heterogeneous human exercise findings; no independent participant sample. | 25 |
| A07 Houttu V, Boulund U, Nicolaou M, Holleboom AG, Grefhorst A, Galenkamp H, et al (2021) | Multiethnic observational cohort | Physical activity and diet both relate to microbial patterns; confounding is relevant. | 3, 26, 28, 29 |
| A08 Min L, Ablitip A, Wang R, Luciana T, Wei M, Ma X (2024) | Systematic review and meta-analysis | Synthesizes exercise studies; underlying interventions and outcomes vary. | Domain context |
| A09 Moitinho-Silva L, Wegener M, May S, Schrinner F, Akhtar A, Boysen TJ, et al (2021) | Randomized exercise intervention | Short-term randomized exercise evidence; group-wide microbial effects were not uniform. | 26, 30 |
| A10 O' Donovan CM, Nori SRC, Shanahan F, Celentano G, Murphy TB, Cotter PD, et al (2025) | Observational athlete study | Reports limited differences across athlete training subgroups. | 28 |
| A11 Verheggen RJHM, Konstanti P, Smidt H, Hermus ARMM, Thijssen DHJ, Hopman MTE (2021) | Exercise intervention in obesity | Exercise improved metabolic measures with modest microbial changes in obesity. | 30 |
| A12 David LA, Maurice CF, Carmody RN, Gootenberg DB, Button JE, Wolfe BE, et al (2014) | Controlled diet intervention | Short-term diet change altered microbial composition; does not validate individual GLAM wording. | 18 |
| A13 Fackelmann G, Manghi P, Carlino N, Heidrich V, Piccinno G, Ricci L, et al (2025) | Large multicohort observational study | Diet-pattern associations in a large cohort; specific item effects remain observational. | 6, 8, 16, 18 |
| A14 Ferrocino I, Di Cagno R, De Angelis M, Turroni S, Vannini L, Bancalari E, et al (2015) | Cross-sectional human diet comparison | Diet-group comparisons using culture/DGGE rather than contemporary sequencing. | Domain context |
| A15 Kohnert E, Kreutz C, Binder N, Hannibal L, Gorkiewicz G, Müller A, et al (2021) | Randomized diet intervention | Randomized vegan versus meat-rich dietary intervention; responses were not universal. | 18 |
| A16 Leeming ER, Johnson AJ, Spector TD, Le Roy CI (2019) | Narrative review | Review of diet intervention duration; background source without independent sample. | Domain context |
| A17 Lkhagva E, Chung HJ, Ahn JS, Hong ST (2021) | Human diet-shift study | Host factors may outweigh a short diet shift; cautions against simple diet attribution. | Domain context |
| A18 Losasso C, Eckert EM, Mastrorilli E, Villiger J, Mancin M, Patuzzi I, et al (2018) | Cross-sectional human diet comparison | Cross-sectional vegan/vegetarian/omnivore comparison; diet groups may differ in other exposures. | 18 |
| A19 Maskarinec G, Hullar MAJ, Monroe KR, Shepherd JA, Hunt J, Randolph TW, et al (2019) | Observational multiethnic cohort | Diet quality associated with microbial diversity/structure; observational evidence. | 12 |
| A20 Maukonen M, Koponen KK, Havulinna AS, Kaartinen NE, Niiranen T, Méric G, et al (2024) | Observational adult cohort | Plant foods, meat and dairy associated with microbial patterns; food-specific directions vary. | 1, 2, 3, 6, 7, 8, 9, 16 |
| A21 Partula V, Mondot S, Torres MJ, Kesse-Guyot E, Deschasaux M, Assmann K, et al (2019) | Cross-sectional diet–microbiota study | Usual food categories associated with microbial composition; observational evidence. | 1, 7, 9, 10, 11, 12, 14, 21 |
| A22 So D, Whelan K, Rossi M, Morrison M, Holtmann G, Kelly JT, et al (2018) | Systematic review and meta-analysis | Fiber interventions show microbial responses but no universal diversity increase. | 3, 4, 5, 17 |
| A23 Trefflich I, Jabakhanji A, Menzel J, Blaut M, Michalsen A, Lampen A, et al (2020) | Cross-sectional study and systematic review | Mixed cross-sectional and review evidence on plant-based diets; avoid universal claims. | 18 |
| A24 Wu GD, Chen J, Hoffmann C, Bittinger K, Chen YY, Keilbaugh SA, et al (2011) | Observational diet-pattern study | Long-term dietary patterns linked to enterotypes; does not isolate fast-food frequency. | 8, 14 |
| A25 Yu D, Nguyen SM, Yang Y, Xu W, Cai H, Wu J, et al (2021) | Observational adult cohort | Diet quality and selected food groups associated with microbial composition. | 1, 2, 5, 7, 9, 10 |
| A26 Jung Y, Tagele SB, Son H, Ibal JC, Kerfahi D, Yun H, et al (2020) | Combined lifestyle-change study | Combined lifestyle change; separate effects of alcohol, diet and activity cannot be inferred. | 13 |
| A27 Rothschild D, Weissbrod O, Barkan E, Kurilshikov A, Korem T, Zeevi D, et al (2018) | Observational host/environment study | Environmental/host context; not a direct test of a single GLAM lifestyle item. | Domain context |
| A28 Benedict C, Vogel H, Jonas W, Woting A, Blaut M, Schürmann A, et al (2016) | Sleep-restriction crossover experiment | Experimental partial sleep deprivation and microbial outcomes; small sample. | 32 |
| A29 Grosicki GJ, Riemann BL, Flatt AA, Valentino T, Lustgarten MS (2020) | Pilot observational sleep study | Global subjective sleep quality and microbiota in a pilot; not item-level validation. | 33, 35 |
| A30 Liu Z, Wei ZY, Chen J, Chen K, Mao X, Liu Q, et al (2020) | Experimental sleep–wake shift | Acute sleep–wake timing change and microbial community; wake time alone not isolated. | 34 |
| A31 Smith RP, Easson C, Lyle SM, Kapoor R, Donnelly CP, Davidson EJ, et al (2019) | Observational sleep physiology study | Sleep physiology associated with microbial diversity; observational evidence. | 32, 36 |
| R01 Vanhaecke T, Bretin O, Poirel M, Tap J (2022) | Observational water-source/intake study | Water intake and source associations; added during revision, not original development evidence. | 15, 19 |
| R02 Suez J, Cohen Y, Valdés-Mas R, Mor U, Dori-Bachash M, Federici S, et al (2022) | Human sweetener intervention | Compounds and individual responses differ; added during revision. | 22 |
| R03 Di Renzo L, Frank G, Pala B, Cianci R, La Placa G, Raffaelli G, et al (2025) | Pilot dietary comparison | Preliminary organic-versus-conventional diet context; added during revision. | 20 |
| R04 Estaki M, Langsetmo L, Shardell M, Mischel A, Jiang L, Zhong Y, et al (2023) | Observational sleep study | Sleep measures and medication covariate; no drug-specific effect established; added during revision. | 37 |

# B Sources that appear related but do not meet the biological evidence question

These are transparent boundary decisions in this retrospective appraisal. They are not represented as members of the unverified historical set of 28 full-text exclusions. Sources used for questionnaire methods may be retained as methodological citations without being counted as studies of lifestyle exposure and gut microbiota outcomes.

| **Citation** | **Why outside the exposure–gut microbiota evidence set** |
| --- | --- |
| Formisano et al. 2024, Stance4Health-FFQ (manuscript ref. 29) | Validated dietary assessment against intake measures; no direct item-specific gut microbiota outcome establishing the GLAM questions. Retained as an assessment framework. |
| Craig et al. 2003, IPAQ (ref. 16) | Physical-activity questionnaire reliability/validity study; no gut microbiota outcome. Retained for measurement context. |
| Buysse et al. 1989, PSQI (ref. 17) | Sleep-quality instrument development; no gut microbiota outcome. Retained for measurement context. |
| Clancy et al. 2021, dietary management of FMT (ref. 30) | Survey of FMT practice and attitudes; relevant to application context, not a study relating an individual lifestyle exposure to measured gut microbiota. |
| Diamond et al. 2014, Delphi reporting (ref. 24) | Consensus-method review; relevant to study design, not to the lifestyle–gut-microbiota evidence question. |

# C Map of all 37 candidate variables

The map distinguishes direct or indirect rationale from measurement context. An item can be relevant metadata without a demonstrated independent microbiota effect. Codes A and R refer to section A; M15, M16, M17 and M29 are manuscript assessment sources, and M26 is the Baxter fiber intervention at manuscript reference 26.

| **Item** | **Candidate variable** | **Sources** | **Interpretation and limit** |
| --- | --- | --- | --- |
| 1 | Fruit consumption frequency | A20 A21 A25 | Exposure-specific human associations; direction is observational and does not establish causation. |
| 2 | Vegetable consumption frequency | A20 A25 | Plant-food and diet-quality context; effects vary across food groups and populations. |
| 3 | Whole grain intake | A07 A20 A22 | Grain and fiber context. Whole-grain-specific effects are not isolated by every cited study. |
| 4 | Dietary fiber intake | A22; M26 | Fiber interventions and synthesis. Supports microbial and metabolic responses, not a universal increase in diversity. |
| 5 | Legume consumption | A25 A22 | Nuts/legumes are assessed together in A25; fiber context in A22. Legume-specific effects are not isolated. |
| 6 | Fermented food consumption | A20 A13 | Fermented dairy and food-associated microbes provide context; not a general intervention test of all fermented foods. |
| 7 | Dairy product consumption | A20 A21 A25 | Dairy-related human associations; direction differs by product and microbial outcome. |
| 8 | Red meat consumption | A13 A20 A24 | Human red-meat or animal-food pattern associations; not evidence for a universal TMAO mechanism. |
| 9 | Processed meat consumption | A20 A21 A25 | Processed-meat exposure is measured in human cohorts; observational associations remain confounded. |
| 10 | Fish consumption | A21 A25 | Fish/seafood is assessed in human cohorts. An omega-3 mechanism is not established by this mapping. |
| 11 | Sugary beverage intake | A21 | Sugary drinks are associated with microbial features in a human cohort; not a causal dysbiosis claim. |
| 12 | Ultra-processed food consumption | A21 A19 | Ready-cooked foods and overall diet quality provide context. A NOVA-defined ultra-processed-food effect is not established here. |
| 13 | Alcohol consumption | A26 | Alcohol is one component of a combined lifestyle change. An independent alcohol effect is not established. |
| 14 | Fast-food consumption frequency | A21 A24 | Fried/ready-cooked foods and long-term dietary patterns provide indirect context; fast-food frequency is not separately validated. |
| 15 | Daily water intake | R01 | Human water-intake associations provide exposure rationale; the observational categories do not validate a GLAM volume threshold. |
| 16 | Probiotic food consumption | A13 A20 | Food-associated microbes and fermented dairy provide context. These findings do not establish probiotic efficacy of the GLAM item. |
| 17 | Prebiotic-rich food consumption | A22; M26 | Fiber and fermentable-substrate evidence; specific foods, dose and outcome require definition. |
| 18 | Plant-based food consumption | A12 A13 A15 A18 A23 | Diet-pattern studies and synthesis. Null or inconsistent findings preclude a universal diversity claim. |
| 19 | Type of drinking water | R01 | Human water-source associations provide exposure rationale; source contrasts are observational and do not establish causation. |
| 20 | Organic food consumption | R03 | Small organic-versus-conventional dietary study; preliminary context, without establishing general superiority of organic foods. |
| 21 | Cooking methods | A21 | Fried and ready-cooked food categories offer indirect context. Cooking method is not isolated from food type or composition. |
| 22 | Type of sweeteners used | R02 | Human intervention evidence for specific non-nutritive sweeteners; effects differ across compounds and people, and do not validate GLAM wording. |
| 23 | Food purchasing location | No direct location-specific source | Purchasing-context metadata; a location-specific microbiota association was not identified in this focused appraisal. |
| 24 | Frequency of vigorous physical activity | A01 A04 A05; M16 | Exercise frequency/intensity context and PA assessment framework. Athlete diet and other confounders matter. |
| 25 | Duration of vigorous activity | A01 A05 A06; M16 | Training-duration context. Supports recording exposure, not a validated dose-response threshold. |
| 26 | Frequency of moderate physical activity | A01 A07 A09; M16 | Moderate/endurance activity context; heterogeneous responses. Expert consensus was not reached for this item. |
| 27 | Walking frequency | M16 | Established PA assessment framework; a walking-specific microbiota association is not established in the mapped publications. |
| 28 | Total weekly physical activity time | A07 A10; M16 | Total/habitual activity context. A10 reports no significant differences across training subgroups. |
| 29 | Sedentary behavior (daily sitting time) | A02 A07; M16 | Active/sedentary comparisons provide human context; daily sitting time is not a validated independent microbiota predictor. |
| 30 | Participation in structured exercise | A01 A05 A09 A11 | Human exercise interventions; null, modest and individual-dependent responses must be retained. |
| 31 | Occupational physical activity | M16 | PA measurement framework; an occupational-activity-specific microbiota association is not established here. |
| 32 | Total hours of sleep per night | A28 A31; M17 | Sleep-restriction experiment and actigraphy associations; small or observational studies do not validate a GLAM outcome. |
| 33 | Sleep onset latency | A29; M17 | PSQI component and global sleep-quality context; a latency-specific microbiota association is not established. |
| 34 | Wake-up time | A30; M17 | Sleep–wake timing experiment and sleep assessment context; wake-up time alone is not isolated. |
| 35 | Subjective sleep quality | A29; M17 | Global PSQI is associated with microbial features in a pilot study; GLAM subjective-quality wording is not independently validated. |
| 36 | Night awakenings | A31; M17 | Wake-after-sleep-onset associations and sleep-disturbance assessment context; no causal item-level validation. |
| 37 | Use of sleep medication | M17 R04 | Sleep-assessment component; medication use was a covariate in a human sleep–microbiota study. This does not demonstrate a medication-specific effect. |

# D Full bibliographic details

A01. Allen JM, Mailing LJ, Niemiro GM, Moore R, Cook MD, White BA, et al. Exercise Alters Gut Microbiota Composition and Function in Lean and Obese Humans. Med Sci Sports Exerc. 2018;50(4):747-757. doi:10.1249/MSS.0000000000001495. PMID 29166320. Also manuscript reference 40.

A02. Bressa C, Bailén-Andrino M, Pérez-Santiago J, González-Soltero R, Pérez M, Montalvo-Lominchar MG, et al. Differences in gut microbiota profile between women with active lifestyle and sedentary women. PLoS One. 2017;12(2):e0171352. doi:10.1371/journal.pone.0171352. PMID 28187199.

A03. Cerdá B, Pérez M, Pérez-Santiago JD, Tornero-Aguilera JF, González-Soltero R, Larrosa M. Gut Microbiota Modification: Another Piece in the Puzzle of the Benefits of Physical Exercise in Health? Front Physiol. 2016;7:51. doi:10.3389/fphys.2016.00051. PMID 26924990.

A04. Clarke SF, Murphy EF, O'Sullivan O, Lucey AJ, Humphreys M, Hogan A, et al. Exercise and associated dietary extremes impact on gut microbial diversity. Gut. 2014;63(12):1913-20. doi:10.1136/gutjnl-2013-306541. PMID 25021423.

A05. Donati Zeppa S, Amatori S, Sisti D, Gervasi M, Agostini D, Piccoli G, et al. Nine weeks of high-intensity indoor cycling training induced changes in the microbiota composition in non-athlete healthy male college students. J Int Soc Sports Nutr. 2021;18(1):74. doi:10.1186/s12970-021-00471-z. PMID 34922581.

A06. Dziewiecka H, Buttar HS, Kasperska A, Ostapiuk-Karolczuk J, Domagalska M, Cichoń J, et al. Physical activity induced alterations of gut microbiota in humans: a systematic review. BMC Sports Sci Med Rehabil. 2022;14(1):122. doi:10.1186/s13102-022-00513-2. PMID 35799284.

A07. Houttu V, Boulund U, Nicolaou M, Holleboom AG, Grefhorst A, Galenkamp H, et al. Physical Activity and Dietary Composition Relate to Differences in Gut Microbial Patterns in a Multi-Ethnic Cohort-The HELIUS Study. Metabolites. 2021;11(12):858. doi:10.3390/metabo11120858. PMID 34940616. Also manuscript reference 41.

A08. Min L, Ablitip A, Wang R, Luciana T, Wei M, Ma X. Effects of Exercise on Gut Microbiota of Adults: A Systematic Review and Meta-Analysis. Nutrients. 2024;16(7):1070. doi:10.3390/nu16071070. PMID 38613103.

A09. Moitinho-Silva L, Wegener M, May S, Schrinner F, Akhtar A, Boysen TJ, et al. Short-term physical exercise impacts on the human holobiont obtained by a randomised intervention study. BMC Microbiol. 2021;21(1):162. doi:10.1186/s12866-021-02214-1. PMID 34078289. Also manuscript reference 42.

A10. O' Donovan CM, Nori SRC, Shanahan F, Celentano G, Murphy TB, Cotter PD, et al. Temporal stability and lack of variance in microbiome composition and functionality in fit recreational athletes. Sci Rep. 2025;15(1):5619. doi:10.1038/s41598-025-88723-9. PMID 39955324.

A11. Verheggen RJHM, Konstanti P, Smidt H, Hermus ARMM, Thijssen DHJ, Hopman MTE. Eight-week exercise training in humans with obesity: Marked improvements in insulin sensitivity and modest changes in gut microbiome. Obesity (Silver Spring). 2021;29(10):1615-1624. doi:10.1002/oby.23252. PMID 34467673.

A12. David LA, Maurice CF, Carmody RN, Gootenberg DB, Button JE, Wolfe BE, et al. Diet rapidly and reproducibly alters the human gut microbiome. Nature. 2014;505(7484):559-63. doi:10.1038/nature12820. PMID 24336217.

A13. Fackelmann G, Manghi P, Carlino N, Heidrich V, Piccinno G, Ricci L, et al. Gut microbiome signatures of vegan, vegetarian and omnivore diets and associated health outcomes across 21,561 individuals. Nat Microbiol. 2025;10(1):41-52. doi:10.1038/s41564-024-01870-z. PMID 39762435. Also manuscript reference 47.

A14. Ferrocino I, Di Cagno R, De Angelis M, Turroni S, Vannini L, Bancalari E, et al. Fecal Microbiota in Healthy Subjects Following Omnivore, Vegetarian and Vegan Diets: Culturable Populations and rRNA DGGE Profiling. PLoS One. 2015;10(6):e0128669. doi:10.1371/journal.pone.0128669. PMID 26035837.

A15. Kohnert E, Kreutz C, Binder N, Hannibal L, Gorkiewicz G, Müller A, et al. Changes in Gut Microbiota after a Four-Week Intervention with Vegan vs. Meat-Rich Diets in Healthy Participants: A Randomized Controlled Trial. Microorganisms. 2021;9(4):727. doi:10.3390/microorganisms9040727. PMID 33807447. Also manuscript reference 46.

A16. Leeming ER, Johnson AJ, Spector TD, Le Roy CI. Effect of Diet on the Gut Microbiota: Rethinking Intervention Duration. Nutrients. 2019;11(12):2862. doi:10.3390/nu11122862. PMID 31766592.

A17. Lkhagva E, Chung HJ, Ahn JS, Hong ST. Host Factors Affect the Gut Microbiome More Significantly than Diet Shift. Microorganisms. 2021;9(12):2520. doi:10.3390/microorganisms9122520. PMID 34946120.

A18. Losasso C, Eckert EM, Mastrorilli E, Villiger J, Mancin M, Patuzzi I, et al. Assessing the Influence of Vegan, Vegetarian and Omnivore Oriented Westernized Dietary Styles on Human Gut Microbiota: A Cross Sectional Study. Front Microbiol. 2018;9:317. doi:10.3389/fmicb.2018.00317. PMID 29556222.

A19. Maskarinec G, Hullar MAJ, Monroe KR, Shepherd JA, Hunt J, Randolph TW, et al. Fecal Microbial Diversity and Structure Are Associated with Diet Quality in the Multiethnic Cohort Adiposity Phenotype Study. J Nutr. 2019;149(9):1575-1584. doi:10.1093/jn/nxz065. PMID 31187868.

A20. Maukonen M, Koponen KK, Havulinna AS, Kaartinen NE, Niiranen T, Méric G, et al. Associations of plant-based foods, red and processed meat, and dairy with gut microbiome in Finnish adults. Eur J Nutr. 2024;63(6):2247-2260. doi:10.1007/s00394-024-03406-x. PMID 38753173. Also manuscript reference 36.

A21. Partula V, Mondot S, Torres MJ, Kesse-Guyot E, Deschasaux M, Assmann K, et al. Associations between usual diet and gut microbiota composition: results from the Milieu Intérieur cross-sectional study. Am J Clin Nutr. 2019;109(5):1472-1483. doi:10.1093/ajcn/nqz029. PMID 31051503. Also manuscript reference 37.

A22. So D, Whelan K, Rossi M, Morrison M, Holtmann G, Kelly JT, et al. Dietary fiber intervention on gut microbiota composition in healthy adults: a systematic review and meta-analysis. Am J Clin Nutr. 2018;107(6):965-983. doi:10.1093/ajcn/nqy041. PMID 29757343. Also manuscript reference 38.

A23. Trefflich I, Jabakhanji A, Menzel J, Blaut M, Michalsen A, Lampen A, et al. Is a vegan or a vegetarian diet associated with the microbiota composition in the gut? Results of a new cross-sectional study and systematic review. Crit Rev Food Sci Nutr. 2020;60(17):2990-3004. doi:10.1080/10408398.2019.1676697. PMID 31631671.

A24. Wu GD, Chen J, Hoffmann C, Bittinger K, Chen YY, Keilbaugh SA, et al. Linking long-term dietary patterns with gut microbial enterotypes. Science. 2011;334(6052):105-8. doi:10.1126/science.1208344. PMID 21885731.

A25. Yu D, Nguyen SM, Yang Y, Xu W, Cai H, Wu J, et al. Long-term diet quality is associated with gut microbiome diversity and composition among urban Chinese adults. Am J Clin Nutr. 2021;113(3):684-694. doi:10.1093/ajcn/nqaa350. PMID 33471054. Also manuscript reference 39.

A26. Jung Y, Tagele SB, Son H, Ibal JC, Kerfahi D, Yun H, et al. Modulation of Gut Microbiota in Korean Navy Trainees following a Healthy Lifestyle Change. Microorganisms. 2020;8(9):1265. doi:10.3390/microorganisms8091265. PMID 32825401.

A27. Rothschild D, Weissbrod O, Barkan E, Kurilshikov A, Korem T, Zeevi D, et al. Environment dominates over host genetics in shaping human gut microbiota. Nature. 2018;555(7695):210-215. doi:10.1038/nature25973. PMID 29489753.

A28. Benedict C, Vogel H, Jonas W, Woting A, Blaut M, Schürmann A, et al. Gut microbiota and glucometabolic alterations in response to recurrent partial sleep deprivation in normal-weight young individuals. Mol Metab. 2016;5(12):1175-1186. doi:10.1016/j.molmet.2016.10.003. PMID 27900260.

A29. Grosicki GJ, Riemann BL, Flatt AA, Valentino T, Lustgarten MS. Self-reported sleep quality is associated with gut microbiome composition in young, healthy individuals: a pilot study. Sleep Med. 2020;73:76-81. doi:10.1016/j.sleep.2020.04.013. PMID 32795890. Also manuscript reference 44.

A30. Liu Z, Wei ZY, Chen J, Chen K, Mao X, Liu Q, et al. Acute Sleep-Wake Cycle Shift Results in Community Alteration of Human Gut Microbiome. mSphere. 2020;5(1):e00914-19. doi:10.1128/mSphere.00914-19. PMID 32051239. Also manuscript reference 45.

A31. Smith RP, Easson C, Lyle SM, Kapoor R, Donnelly CP, Davidson EJ, et al. Gut microbiome diversity is associated with sleep physiology in humans. PLoS One. 2019;14(10):e0222394. doi:10.1371/journal.pone.0222394. PMID 31589627. Also manuscript reference 43.

R01. Vanhaecke T, Bretin O, Poirel M, Tap J. Drinking Water Source and Intake Are Associated with Distinct Gut Microbiota Signatures in US and UK Populations. J Nutr. 2022;152(1):171-182. doi:10.1093/jn/nxab312. Also manuscript reference 49.

R02. Suez J, Cohen Y, Valdés-Mas R, Mor U, Dori-Bachash M, Federici S, et al. Personalized microbiome-driven effects of non-nutritive sweeteners on human glucose tolerance. Cell. 2022;185(18):3307-3328.e19. doi:10.1016/j.cell.2022.07.016. Also manuscript reference 50.

R03. Di Renzo L, Frank G, Pala B, Cianci R, La Placa G, Raffaelli G, et al. Effects of Italian Mediterranean Organic Diet on the Gut Microbiota: A Pilot Comparative Study with Conventional Products and Free Diet. Microorganisms. 2025;13(7):1694. doi:10.3390/microorganisms13071694. Also manuscript reference 51.

R04. Estaki M, Langsetmo L, Shardell M, Mischel A, Jiang L, Zhong Y, et al. Association of Subjective and Objective Measures of Sleep With Gut Microbiota Composition and Diversity in Older Men: The Osteoporotic Fractures in Men Study. J Gerontol A Biol Sci Med Sci. 2023;78(10):1925-1932. doi:10.1093/gerona/glad011. Also manuscript reference 52.

Additional assessment/context sources: Willett W. Nutritional Epidemiology. 3rd ed. Oxford: Oxford University Press; 2013; Craig CL et al. Med Sci Sports Exerc. 2003;35:1381–1395; Buysse DJ et al. Psychiatry Res. 1989;28:193–213; Formisano A et al. Nutrients. 2024;16:4064; Baxter NT et al. mBio. 2019;10:e02566-18. Complete entries are retained in the manuscript bibliography.
